# How Geography and Climate Shaped the Genomic Diversity of Italian Local Cattle and Sheep Breeds

**DOI:** 10.3390/ani12172198

**Published:** 2022-08-26

**Authors:** Gabriele Senczuk, Andrea Criscione, Salvatore Mastrangelo, Filippo Biscarini, Donata Marletta, Fabio Pilla, Denis Laloë, Roberta Ciampolini

**Affiliations:** 1 Dipartimento di Agricoltura, Ambiente e Alimenti, Università del Molise, 86100 Campobasso, Italy; 2 Dipartimento Agricoltura, Alimentazione e Ambiente, University of Catania, 95131 Catania, Italy; 3 Dipartimento Scienze Agrarie, Alimentari e Forestali, University of Palermo, 90128 Palermo, Italy; 4 CNR (Consiglio Nazionale delle Ricerche), Istituto di Biologia e Biotecnologia Agraria, 20133 Milan, Italy; 5 GABI, AgroParisTech, INRAE, Université Paris-Saclay, 78350 Jouy-en-Josas, France; 6 Dipartimento di Scienze Veterinarie, Università di Pisa, 56124 Pisa, Italy

**Keywords:** cattle, sheep, genome, cilmate, geography, redundancy analysis

## Abstract

**Simple Summary:**

In this paper, we study the inter-relationships among geography, climate, and genetics in Italian local cattle and sheep breeds. In terms of genetic diversity, geography (latitude and longitude) appears to play a larger role in sheep (26.4%) than that in cattle (13.8%). Once geography is accounted for, 10.1% of cattle genomic diversity and 13.3% of that of sheep are attributable to climatic effects. Stronger geographic effects in sheep can be related to a combination of higher predomestication genetic variability together with biological and productive specializations. The climate alone seems to have had less impact on the current genetic diversity in both species even if climate and geography are greatly confounded. Results confirm that both species are the result of complex evolutionary histories triggered by interactions between human needs and environmental conditions.

**Abstract:**

Understanding the relationships among geography, climate, and genetics is increasingly important for animal farming and breeding. In this study, we examine these inter-relationships in the context of local cattle and sheep breeds distributed along the Italian territory. To this aim, we used redundancy analysis on genomic data from previous projects combined with geographical coordinates and corresponding climatic data. The effect of geographic factors (latitude and longitude) was more important in sheep (26.4%) than that in cattle (13.8%). Once geography had been partialled out of analysis, 10.1% of cattle genomic diversity and 13.3% of that of sheep could be ascribed to climatic effects. Stronger geographic effects in sheep can be related to a combination of higher pre-domestication genetic variability together with biological and productive specificities. Climate alone seems to have had less impact on current genetic diversity in both species, even if climate and geography are greatly confounded. Results confirm that both species are the result of complex evolutionary histories triggered by interactions between human needs and environmental conditions.

## 1. Introduction

Since domestication, livestock species have adapted to abiotic factors that characterized their cradle of origin and subsequently their different breeding areas. Farmers have directed the evolution of livestock species in response to their production needs, and according to the landscape and bioclimatic factors at different latitudes [1,2].

An extensive range of environmental diversity characterizes Italy spanning from the Alps to the Mediterranean climate of the south and the isles [3]. This ecological richness pairs with a wide reservoir of genetic resources of natural and livestock species. Although specialized breeds play a pre-eminent role, several local breeds are reared on the Italian territory that have evolved over the centuries through ancient and recent genetic and demographic events [4,5]. This biodiversity has shaped animal genomes and has adapted to different environments, where it plays a crucial socioeconomic role by contributing to the conservation of the landscape [6], the exploitation of traditional products [7] and consequently to meeting the needs of local communities.

Over the centuries, orographic and climatic conditions have undergone sometimes drastic changes, and conditioned the availability of natural and agricultural resources and, consequently, the farming systems. The reduction in atmospheric precipitation, the increase in temperature, and the decrease in green areas all drastically affect animal production [8,9]. The ability to adapt to climatic variability varies between species and breeds, and relies on their plasticity, defined as the evolutionary adaptation to environmental variation [10,11].

The issue of climatic variability in relation to livestock farming is increasingly present in scientific studies. The climate could adversely affect the health of livestock, and farmers are increasingly aware of the fact that managing the welfare of their animal herds is one of the main factors in maintaining high production levels [11].

In the last decade, Italian cattle and sheep breeds have been widely studied within the framework of the BOVITA [5] and BIOVITA [12] consortia. Capitalizing on the genomewide data available from single-nucleotide polymorphism (SNP) genotyping, relevant aspects such as (i) genetic diversity, (ii) between-breed relationships, (iii) population structure, (iv) quantitative trait loci and genotype–phenotype associations, and (v) geographical patterns have been investigated in local breeds of both cattle [5,13,14,15] and sheep [4,16,17,18].

Since Cavalli-Sforza advocated the use of factorial analysis, namely, principal component analysis (PCA), to decipher the population structuring of genetic diversity [19], these approaches have been widely adopted in genetic population studies. A wide range of factorial methods were used to address the spatial structuring of genetic diversity through spatial PCA [20,21,22], to discriminate among populations [23] and to model genetic diversity in relation to breeds [20]. Other sources of variation, such as the environment or bioclimatic variables, have also long been considered through the use of redundancy analysis [24,25,26,27].

The BOVITA and BIOVITA consortia provide genomic data for different livestock species that originated in the same geographical region, thus enabling to compare the degree of structuration due to geography and/or climate in different species in the same region. This article aims at deciphering the genomic structure of local Italian cattle and sheep breeds in relation to geography and climate. More specifically, the respective roles of geography and climate in shaping genomic structure were assessed through factorial analysis, in particular redundancy analysis. In detail, bioclimatic variables were fed into PCA to obtain a compressed description of Italian climatic diversity summarized in a few dimensions. The most informative principal components were then used as proxies (variables) for the climate. For each species, after PCA on SNP allele frequencies averaged by breeds, redundancy analysis was performed to investigate the way in which geography and climate shaped genomic diversity: breed allele frequencies were modelled as a function of (a) geography (latitude and longitude) and (b) climate (proxy variables from PCA), with geography as a covariable.

## 2. Materials and Methods

### 2.1. Available Data

**Figure 1:**
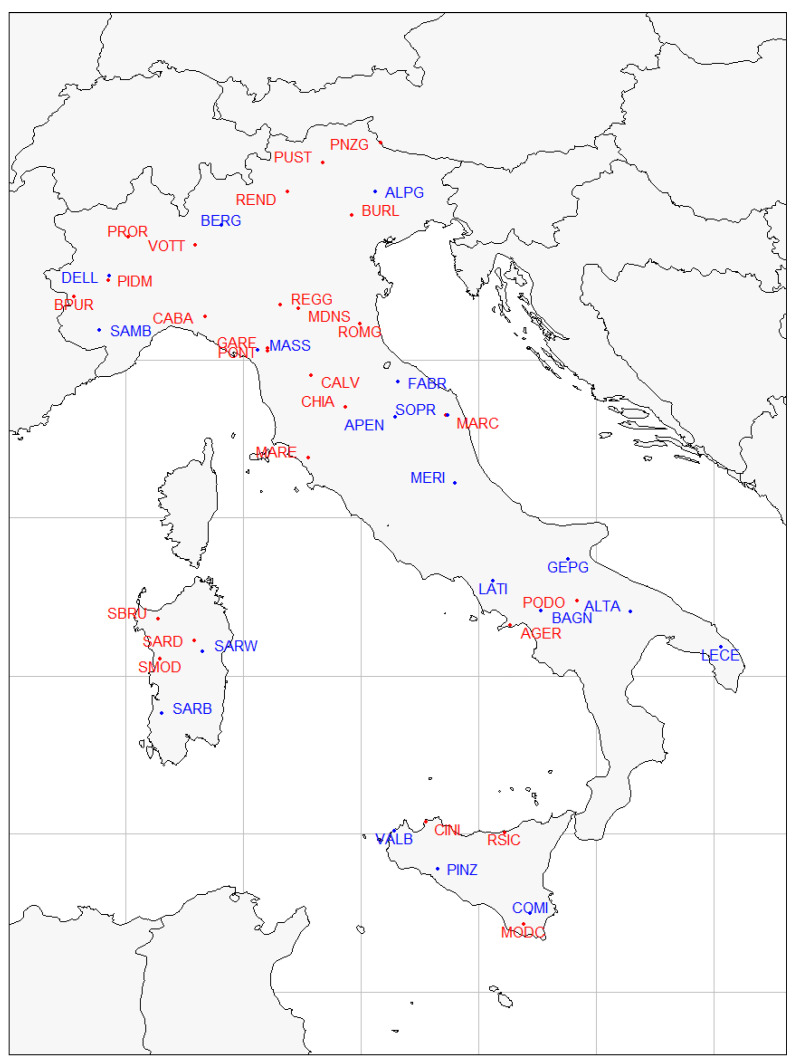
Distribution of cattle and sheep breeds on the map of Italy. Locations correspond to the original geographical cradle of the breed. Points and labels colored by species: bovine in red, ovine in blue.

For this study, we used already published data from previous Italian national research projects: SNP genotypes on 30 cattle breeds from the Bovita consortium, and 23 sheep breeds from the Biovita consortium (Table 1). Full details on these breeds can be found in Mastrangelo et al. [5] for cattle, and in Ciani et al. [12] for sheep. Geographical coordinates were available for each breed and were used to obtain climatic variables from the the Climond database (https://www.climond.org/, accessed on 13 May 2022). The geographical distribution of cattle and sheep breeds on the Italian territory is shown in Figure 1.

### 2.2. Sample Collection and SNP Genotypic Data

#### 2.2.1. Bovine Data

A total of 26 Italian cattle breeds were selected. For all animals, genotypic data from the Illumina BovineSNP50 v2 BeadChip array were retrieved for analysis. The genotypic data came from a previous study [5]. On the basis of the distribution of missing values, we removed samples with more than 6000 missing SNPs, and SNPs with >50 missing values. In addition, we excluded nonautosomal SNPs and SNPs with minor allele frequency (MAF) < 0.05. The remaining missing values were imputed with the median frequency of the corresponding SNPs. After filtering, 36,723 SNPs remained for analysis on 566 samples from 26 local Italian cattle breeds.

#### 2.2.2. Ovine Data

A total of 19 Italian sheep breeds were selected. For all animals, genotypic data from the Illumina OvineSNP50 BeadChip array were retrieved for analysis. The genotypic data came from a previous study [12]. On the basis of the distribution of missing values, we removed samples with more than 8000 missing SNPs and SNPs with more than 100 missing values. Moreover, we excluded nonautosomal SNPs and SNPs with MAF <0.05. The remaining missing values were imputed with the median frequency of the corresponding SNPs. After filtering, 36,611 SNPs remained for analysis on 399 samples from 19 local Italian sheep breeds.

### 2.3. Climatic Variables

Using the GPS coordinates associated to each breed, 35 climatic covariables (from Bio01 to Bio35) were extracted from the Climond database (https://www.climond.org/, accessed on 13 May 2022 [28]) with a resolution of 30’: 11 variables on temperature, and 8 variables each for moisture, radiation, and precipitation (Supporting Table S1). These bioclimatic variables reflect measurable information on annual, weekly, and seasonal temperature, soil moisture, radiation, and precipitation, and are used as a proxy to describe the climate [29]. More specifically, these variables summarize the climatic conditions between 1961 and 1990 in the form of raster data (grids of cells with climate values) at about 19 km spatial resolution [28].

### 2.4. General Statistical Approach

In this paper, we used a series of multivariate statistical techniques to analyze geographic, climatic, and genomic data in Italian sheep and cattle breeds. We first looked at the 35 climatic variables to uncover patterns, and estimate their mutual relationships and how they correlate with geography. We then added genomic data separately for cattle and sheep to study the inter-relations among geography, climate, and genetics. Genetics and the climate were also studied in models that included the effect of geography, thereby allowing for isolating the climate and genetics from specific geographical locations. All these analyses were performed using a supervised version of principal component analysis (PCA), namely, redundancy analysis (RDA) and the associated partial redundancy analysis. All data-processing and statistical analyses were performed with the R environment for statistical programming [30]. Specific R packages are mentioned in the relevant sections.

### 2.5. Redundancy Analysis

RDA combines a linear model and principal component analysis to model multivariate response data by regressing a matrix of variables to be explained (response matrix) against a matrix of explanatory or independent variables. This analysis computes orthogonal axes that are linear combinations of independent variables that best explain variation in response data (see details in [25,31]). The eigenvalues of ordination axes measure the amount of variance in the response matrix explained by the independent variables. The total variance may be partitioned into constrained (model) and unconstrained (residuals) components.

Partial redundancy analysis (partial RDA) provides a way of analyzing residual variation after relevant covariables are partialled out by multivariate regression. Total variance is partitioned into conditioned (covariables), constrained (model), and unconstrained (residuals) components. ANOVA-like permutation (PERMANOVA [32]) tests are performed to assess the global significance of the models, the significance of each included variable, and the significance of the orthogonal axes. Permutational tests are based on the empirical distribution function (EDF) of the test statistics, and produce more robust and reliable results compared to traditional testing approaches (no assumptions on the shape of the distribution). Even if RDA approaches are less sensible to the p>>n problem, and the R *vegan* implementation produces correct levels of Type I error [31], RDA must be associated with testing procedures to check the significance of the results and help in discarding spurious cases [33]. Analyses were performed with the R *vegan* package (RDA and partial RDA [34]) and the R *ade4* package [35].

### 2.6. Specific Statistical Analyses

The following analyses were performed:

#### 2.6.1. Climatic Variability

PCA was performed on the 35 Climond bioclimatic variables in order to obtain a view of the overall variability in the Italian climate and reduce the set of 35 original bioclimatic variables to a few synthetic variables to be used as climatic proxies in subsequent analyses. Geographical coordinates (longitude and latitude) were included in the analysis as supplementary variables. Supplementary variables are not used in the analysis, but can later be projected onto the different principal components.

#### 2.6.2. Association of Climate to Geography

Associations between climate and geography were studied with RDA, where the 35 bioclimatic variables were modelled with respect to the geographical coordinates.

#### 2.6.3. Breed Genomic Variability

Genomic data were the average breed allelic frequencies. Allelic frequencies were previously standardized as follows: if $p_{i}$ is the overall allelic frequency of the $i_{th}$ SNP, then the individual allelic frequencies are divided by ${\left(p_{i}\cdot \left(1-p_{i}\right)\right)}^{\frac{1}{2}}$. This standardization allows for interpreting the inertia of the data table in terms of $F_{st}$ [36,37]. In the case of biallelic SNPs, $F_{st}$ was equal to the data table inertia divided by the number of SNPs [22]. A first PCA was performed on the breed-averaged allelic frequencies in order to obtain an overall picture of the genomic variability at the breed level.

#### 2.6.4. Association of Genome to Climate and Geography

In order to study the relative impact of geography and climate on the genomic diversity of Italian cattle and sheep breeds, two RDA models were run: (i) an RDA modelling of the genomic diversity with respect to the geographical coordinates: genome∼geography (latitude + longitude + latitude × longitude); (ii) a partial RDA modelling of the genomic diversity with respect to climate after partialling out the geography: genome∼climate (Clim1 + Clim2) + condition (latitude + longitude), where Clim1 and Clim2 are the first two climatic principal components; the covariables included in Condition() were partialled out.

## 3. Results and Discussion

### 3.1. Principal Component Analysis of Climatic Data

**Figure 2:**
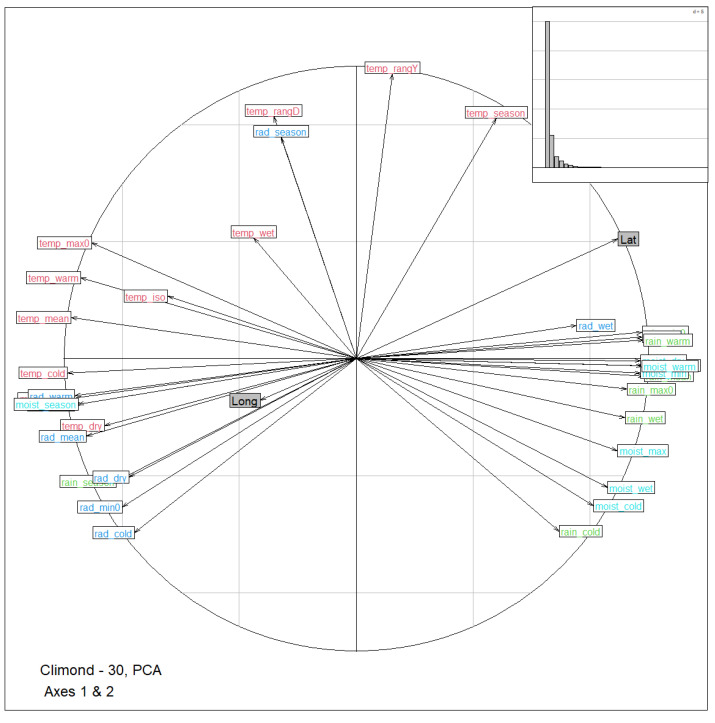
Correlation circle representation of the bioclimatic variables in the first two dimensions of the principal component analysis (PCA) of Climond data. Variables are colored according to their type (deep blue: radiation, green: rain, red: temperature, light blue: moisture). Labels are explained in Table S1. Latitude and longitude were projected as supplementary variables and are shown in grey. Top right within the insert is the corresponding scree plot (eigenvalues, bar plot).

Results from PCA on the 35 bioclimatic variables show that the first two principal components accounted for 70.2% and 16.1% of the total variance for a combined 86.3%. Figure 2 shows the corresponding circle of correlations between the first two components and the bioclimatic variables. Latitude and longitude were projected as supplementary variables onto the correlation circle. Climatic variable loadings for the first two axes are plotted in Supplementary Figures S1 and S2. The first principal component was mainly produced from the opposition between the average values of temperature and radiation on the one hand, and moisture and rain on the other hand. This component was also strongly associated with latitude (r=−0.85), and weakly with longitude (r=−0.31). The second component was associated with the seasonality and the range of radiation and temperature (annual and dairy temperature range, seasonality of temperature and radiation) against hivernal values for rain and moisture. This component was less related to geography (r=−0.49 and r=−0.26 for latitude and longitude, respectively).

**Figure 3:**
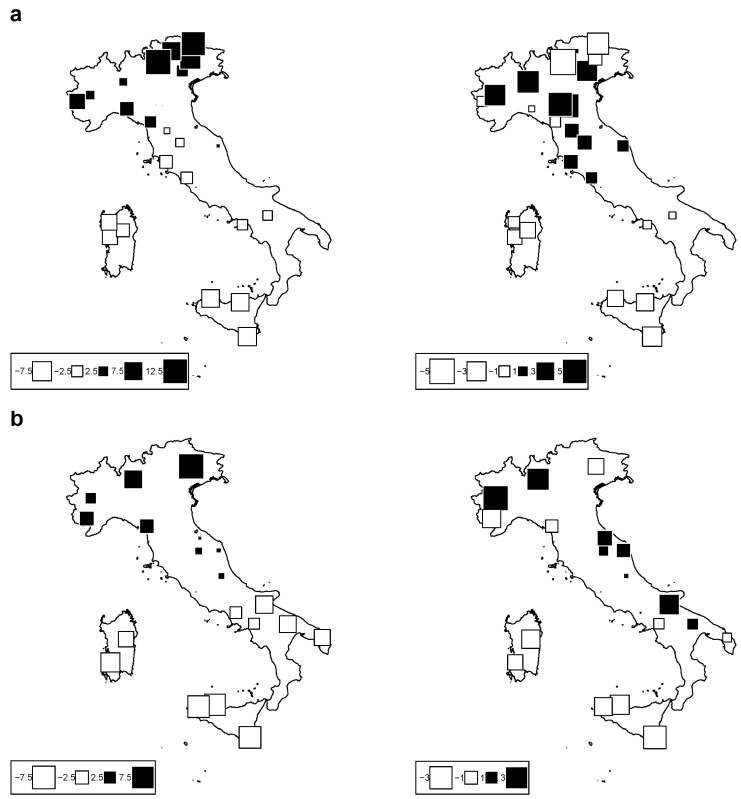
Bubble-plot maps of the climatic PCA scores for: (**a**) cattle breeds (**top**) and (**b**) sheep breeds (**bottom**) for Axis 1 (**left**) and Axis 2 (**right**). Black square bubbles indicate positive score values; white square bubbles indicate negative square values; square size is proportional to the absolute score values.

Figure 3 is a bubble plot map of the PCA scores for cattle (Figure 3a) and sheep (Figure 3b) breeds along the first two axes; breeds are located at their original cradle. Black square bubbles represent positive score values, and white bubbles negative values; the square size is proportional to the absolute score value. On the left, the bubble plot for Axis 1 shows a strong trend of the first climate component with latitude, as already indicated by the strong correlation between axis 1 and latitude. The bubble plot for Axis 2 indicates a separation between east and west in the case of cattle in the Italian Alps; on the other hand, sheep breeds show an admixed correlation trend towards the second climate component. According to these results, we kept the first two components as proxy variables to synthesize the whole set of bioclimatic variables. We define these two proxy variables as “Clim1” and “Clim2” throughout the text.

The redundancy analysis that modelled the climate as a function of latitude and longitude (Table 2) shows that latitude was strongly associated with climate with an inertia ratio equal to 59% and *p*-value = 0.001.

### 3.2. Genomic Diversity of Cattle Breeds

**Figure 4:**
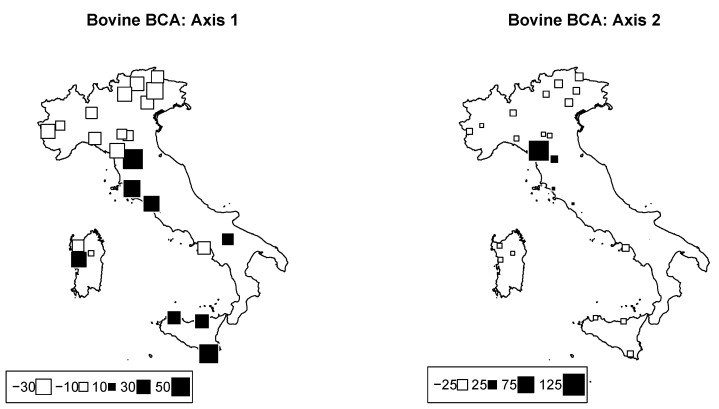
Bovine genome between−breed analysis. Bubble plots of the values of the bovine breeds scores for the first two principal components (**left**: PC1; **right**: PC2). Breeds are located at their original geographical cradle. Black bubbles have positive values and white ones negative values; square size is proportional to the score absolute values.

PCA on genomic cattle data was performed. The overall $F_{st}$ among breeds was 0.177. Bubble plots were drawn for Axes 1 and 2 in order to visually assess the geographic structuration of the breeds (Figure 4). The first two components accounted for 11.9% and 8.3% of the total inertia, respectively.

Interestingly, the first axis clearly discriminated all breeds that belonged to the so-called Podolian trunk. These breeds have a complex evolutionary history, recently dissected using genomewide SNPs, and probably belong to a secondary wave of migration through both the Danubian and Mediterranean routes after the arrival of indicine cattle in the Middle East [38]. On the other hand, the second axis mainly distinguishes some breeds from Central Italy, and in particular two breeds from Tuscany, the Pontremolese, and Garfagnina. These outcomes might have been caused by local genetic drift followed by inbreeding to which these neighboring breeds have been subjected. Indeed, recent assessments on the genetic diversity of global cattle breeds highlighted a marked level of molecular inbreeding ($F_{ROH}$) in the Garfagnina and Pontremolese breeds, probably caused by the recent numerical decline of these populations [5,18]. As a matter of fact, our results from PCA confirm the genetic structuring of Italian cattle populations according to the breeds’ origins and evolutionary histories, as also underlined in previous works.

#### 3.2.1. Redundancy Analysis according to Geography in Cattle

**Figure 5:**
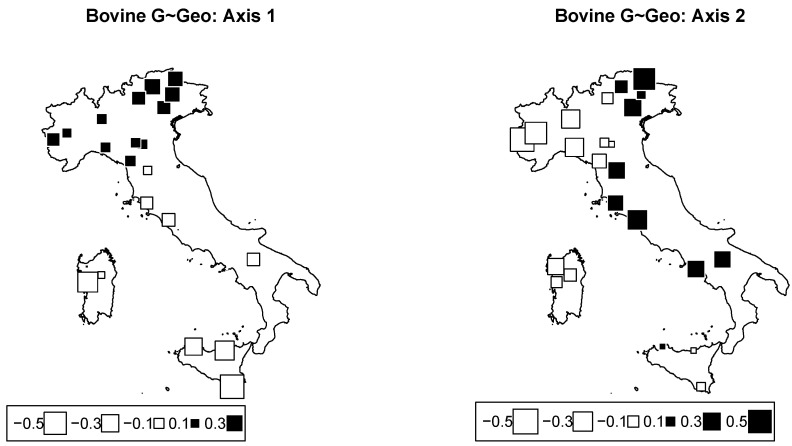
Cattle genome redundancy analysis (RDA) according to the model genome ∼ geography. Bubble plots of the values of the breed scores for the first two principal components (**left**: PC1; **right**: PC2). Black square bubbles are positive; white square bubbles are negative; square size is proportional to the score absolute values.

Redundancy analysis according to the model of $\left[\mathrm{genome}∼\mathrm{latitude}\cdot \mathrm{longitude}\right]$ was performed. Table 3 gives the inertial percentages due to different effects. Inertia due to the model was 13.8% of the total inertia. Axes 1 and 2 accounted for 7.5% and 4.6% of the total inertia, respectively. Only Axis 1 was significant, with *p*-value = 0.014. Figure 5 represents bubble plots (Axes 1 and 2) corresponding to this analysis. The bubble plots replicate the structuration observed with climatic data (Figure 4): the first axis was mainly built by the latitude, while the second one isolated northwestern breeds from northeastern breeds.

Studies that deal with genomic variability and geographic cline have shown a general trend between the latitude and the variability of genetic resources, and cattle is no exception [39]. According to the first axis, a major genetic discontinuity related to the geography is observable in the Northern Apennines. From this perspective, the trend in which the analyzed cattle breeds were distributed according to the latitude on the Italian territory can be partly explained by the orographic conformation, the availability of water, and the north–south temperature gradient. On the other hand, the second axis mostly reveals a genetic discontinuity in Alps along the longitudinal axis. This genetic repartition according to geographic origin had already been highlighted for alpine breeds especially when comparing breeds from the western and eastern Alps [15].

#### 3.2.2. Redundancy Analysis according to Climate Conditioned for Geography in Cattle

**Figure 6:**
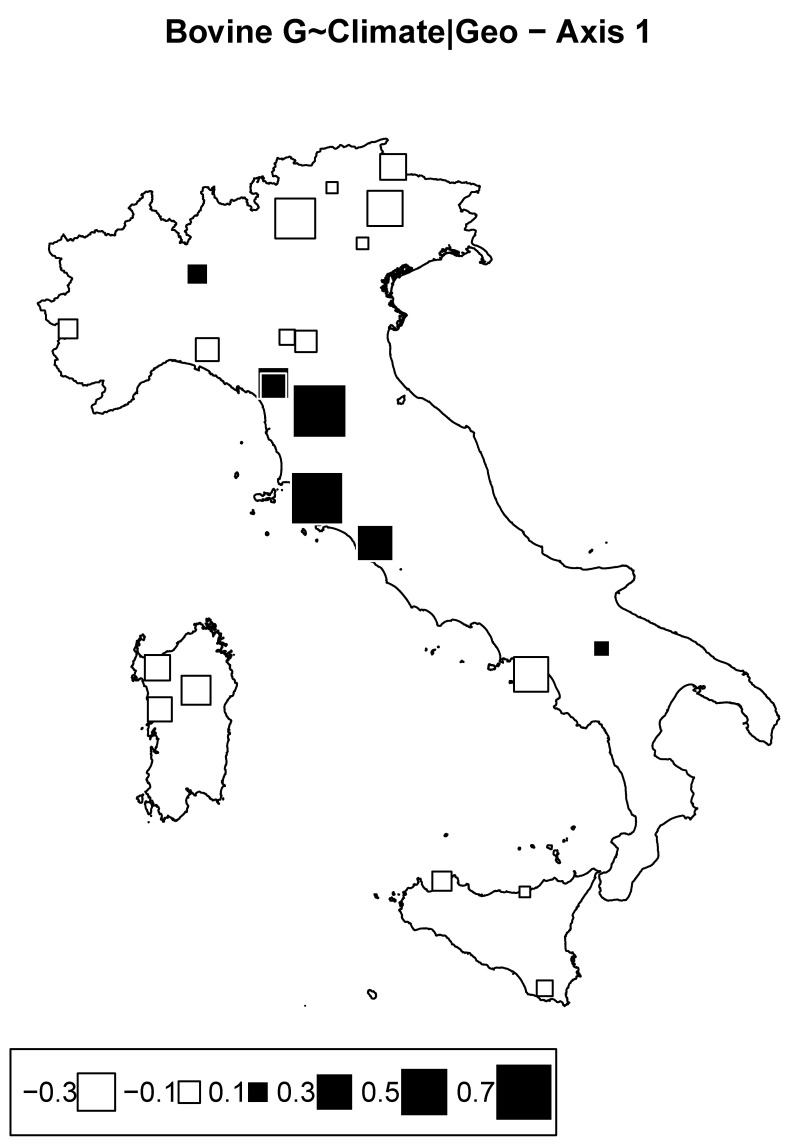
Bovine genome redundancy analysis (RDA) according to the model genome ∼ climate partialled out for geography. Bubble plots of the breed scores for the first principal component. Black square bubbles are positive; white square bubbles are negative; square size is proportional to the score absolute values.

We consider the model of genome ∼ climate + condition (geography), where climate is described by the two principal components issued from the PCA on climatic data, and geography is described by the geographical coordinates. Climate accounted for 10.4% of the inertia. Out of the two RDA axes, only the first was near a significant threshold of 10% with *p*-value = 0.09 (significant, with 7.3% of the total inertial). The corresponding bubble plot is reported in Figure 6, showing an isolation of Tuscany breeds against others.

These results are rather difficult to interpret, as we cannot speculate about specific environmental conditions explained by the Clim2 proxy that might have impacted the genetic structure of these breeds from Central Italy. Moreover, we cannot completely rule out other demographic factors in generating such patterns. Indeed, signs of inbreeding and genetic drift, especially in the Garfagnina and Pontremolese breeds, were documented [14].

### 3.3. Genomic Diversity of Sheep Breeds

**Figure 7:**
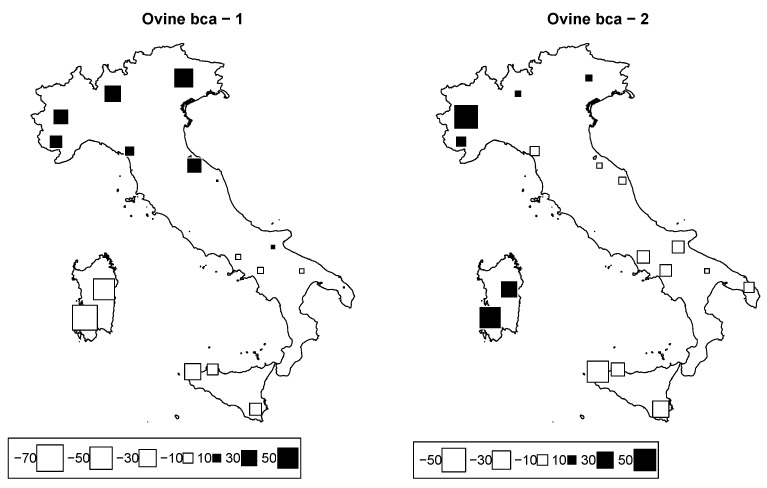
Ovine genome between-breed analysis. Bubble plots of the bovine breeds scores for the first two principal components (**left**: PC1; **right**: PC2). Breeds are located at their original geographical cradle. Black square bubbles are positive; white square bubbles are negative; square size is proportional to the score absolute values.

The general $F_{st}$ among sheep breeds was 0.14. PCA results are visualized through bubble plots in order to assess the geographic structure of the breeds (Figure 7). Of the initial 19 selected breeds, Appenninica and Merinizzata were removed because of a ≥20% SNP missing rate. However, the removal of these two breeds did not affect the subsequent results. The first two axes accounted for 22.5% of the total inertia (12.5% and 10.0% of the inertia for Axes 1 and 2, respectively), and isolated north from south (Axis 1) and west from east (Axis 2). The sheep genetic distribution along the two axes, as reported in Figure 1, highlights the presence of a genetic latitudinal cline in the Italian peninsula, as suggested by the presence of unstable groups between the two axes (Figure 7). This genetic structure parallels several previous works that highlighted the presence of a latitudinal gradient; the differentiation of the two Sardinian breeds (SARB and SARW) with respect to all other peninsular breeds was probably due to the combined effect of prolonged isolation with sporadic introgression with the wild mouflon [12].

#### 3.3.1. Redundancy Analysis According to Geography in Sheep

**Figure 8:**
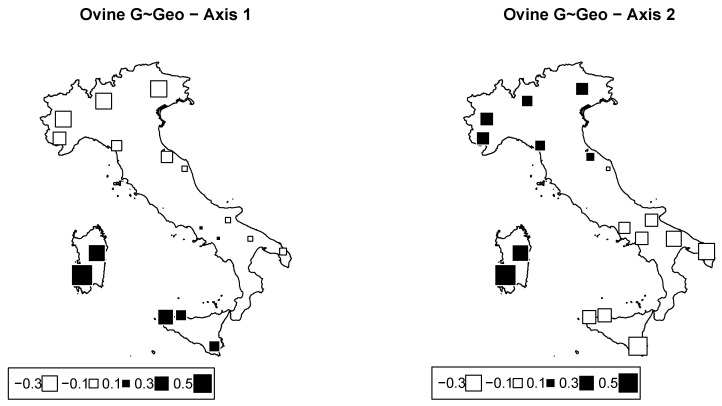
Ovine genome redundancy analysis (RDA) according to the model genome ∼ geography. Bubble plots of the values of the sheep breeds scores for the first two principal components (**left**: PC1; **right**: PC2). Black square bubbles are positive; white square bubbles are negative; square size is proportional to the score absolute values.

From RDA, the proportion of inertia owing to geography was 0.263, i.e., about twice that estimated in cattle. The first two axes from RDA were significant (p=0.001 and 0.029 for Axes 1 and 2, respectively), and they accounted for 11.5% and 8.1% of the total inertia. Corresponding bubble plots are plotted in Figure 8. Table 4 indicates the percentage of inertia due to latitude, longitude, and their interaction. All were significant (p<0.01).

The first axis mainly distinguishes breeds from the two islands (Sicily and Sardinia) and the Laticauda and Bagnolese breeds from all other breeds. The genetic relationship between Sicilian and Sardinian breeds was highlighted in previous works [40,41] as a result of the historical admixture among them, in particular the contribution of Sarda to the origin of the VDB breed. The second axis separates Sardinian and northern breeds from all southern and Sicilian breeds. These results basically confirm the genomic PCA that highlighted a latitudinal gradient and genomic differences of the insular breeds of Sicily and Sardinia. The geographic relationships between the two autochthonous breeds, Laticauda and Bagnolese, with breeds from Sicily was probably the result of a rather recent crossbreeding event with fat-tailed sheep from North Africa, which also influence Sicilian sheep populations [42].

#### 3.3.2. Redundancy Analysis According to Climate Conditioned for Geography in Sheep

**Figure 9:**
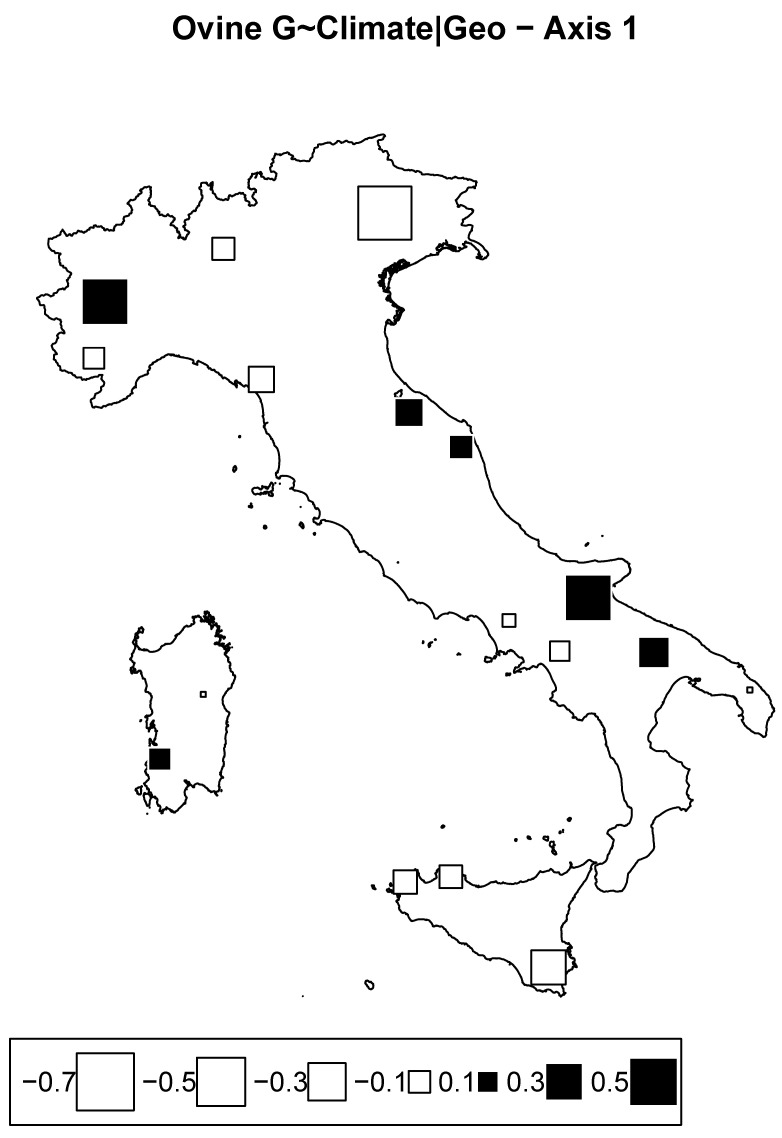
Ovine genome redundancy analysis (RDA) according to the model genome ∼ climate, partialled out for geography. Bubble plots of the values of the sheep breed scores for the first principal component. Black square bubbles are positive; white square bubbles are negative; square size is proportional to the score absolute values.

RDA taking into account climate variables corrected for geography explained 7.5% of the total inertia. However, in this case, both axes show nonsignificant values. The bubble plot as shown in Figure 9 depicts a prominent effect of climatic conditions through the east–west axis. Although the values were not significant and should thereby be taken with caution, the picture indicates a substantial differentiation of Tyrrhenian breeds from Adriatic breeds. Differences in climatic conditions and environments between the Adriatic and Tyrrhenian coasts are undoubtedly known [43]. These may have had repercussions on the genetic structure of sheep populations, especially when considering that sheep and more generally small ruminants appear to be more susceptible to climatic variability, as they are extensively reared and strongly dependent on pasture conditions. However, we cannot exclude a possible hidden factor due to the traditional long-range seasonal transhumance in molding the observed pattern. Indeed, especially for Central Italy, this practice may have increased genetic flow within the Adriatic and Tyrrhenian populations while reducing the genetic exchange between them.

### 3.4. Cattle and Sheep Genetic Diversity along the Climatic and Geographic Dimensions

The decomposition of inertia into geography (combination of latitude and longitude) and climate, as summarized by the first two PC of the climatic PCA, is shown in Table 5. This decomposition was sequential: the geography was partialled out before analyzing the climate effect. In other words, 10.1% of the cattle genomic diversity and 13.3% of that of sheep could be assigned to climatic effects once the geography effects had been taken into account. Geography appears to be more important in sheep (26.3%) than in cattle (14.7%). The prominent effect of geography in molding the genetic architecture in cattle and sheep was shown in previous works [12,14,15]. Other than that, the partition of the inertia was similar in both species, with similar $F_{st}$ values (Table 5).

The genomic architecture of these two species is complex due to a combination of several factors, including the heterogeneous predomestication genomic pool, the European diffusion via different spatiotemporal migration routes, and the subsequent admixture with both wild counterparts (*Bos primigeneus* for cattle, mouflon for sheep) and local populations [38,44,45,46].

However, the fact that we found higher values of inertia in sheep than those in cattle could be related to several factors. First, an active role of different evolutionary trajectories during the domestication diffusion processes cannot be ruled out. For example, compared to other domestic species such as goats, current sheep breeds derive from an important cradle of genetic heterogeneity [47]. Second, although both species with their corresponding breed diversity have a longstanding history in the Italian peninsula, biological and productive specificities render the farming of the two species very different. In this context, traditional extensive sheep farming centred on vertical and horizontal transhumance may have reduced the genetic flow between distant geographic areas; this, in turn, increased isolation and genetic discontinuities while generating a latitudinal cline, especially in central Apennine areas where long-range transhumance has historically played a dominant role. On the other hand, the weaker geographic structure of cattle breeds seems to reflect the differential diffusion across the Italian Peninsula, confirming the Podolian cattle as the main genetically distinct group of breeds.

Concerning the climatic dimension, we found no significant values in either species except for Clim2 (second PC from the climatic PCA), which isolated Tuscany breeds from the others. However, when looking at the absolute values, in this case, sheep also showed slightly higher inertia, which might reflect increased susceptibility to environmental factors in shaping genetic diversity in this species [48]. In particular, considering the climatic dimension through bubble plots, sheep seem to be more influenced by climatic differences along the longitudinal west–east axis; for cattle, we did not find a pattern that could be easily attributed to climate, as the Tuscan breeds were the only strongly differentiated ones.

## 4. Conclusions

Cattle and sheep have always been an integral and important part of human society, as cattle provide large amounts of milk and meat per reared unit, and sheep can fruitfully exploit large marginal areas. Nevertheless, a comparative framework aimed at disentangling the effect of climate and geography on the genome of these two species is still lacking. The availability of genomic data from local Italian sheep and cattle breeds allowed for us to propose a comparison procedure for the evaluation of the spatial–climatic dimensions of the overall genetic diversity. This procedure was based on the description of space with geographic coordinates, and on a bivariate summary of climatic variables through PCA on bioclimatic data. Lastly, the genetic structure was examined with redundancy analysis as a function of the above-mentioned two factors of geography and climate. This spatial modelling is rather simple and limited to linear trend effects. However, it allowed for us to build a common spatial reference among species, and then to appreciate how or if the environment generally had differently shaped the genetic diversity in these two species. The outlined results highlight some critical issues when studying highly structured populations, and can hinder attributing the observed genetic structure purely to climate even after partialling out geography. Further studies using either other livestock species or different geographic areas are desirable in order to have a better overview of how climate and geography might have impacted the current biodiversity at the genomic level.

## Figures and Tables

**Table 1:** List of Italian cattle and sheep breeds used in this study with the full names and abbreviations used in the article. Cattle breeds were from the BOVITA consortium; sheep breeds were from the BIOVITA consortium.

CATTLE		SHEEP	
Agerolese	AGER	Alpagota	ALPG
Bará-pustertaler	BPUR	Altamurana	ALTA
Burlina	BURL	Appenninica	APEN
Cabannina	CABA	Bagnolese	BAGN
Calvana	CALV	Bergamasca	BERG
Chianina	CHIA	Comisana	COMI
Cinisara	CINI	Delle Langhe	DELL
Garfagnina	GARF	Fabrianese	FABR
Marchigiana	MARC	Gentile di Puglia	GEPG
Maremmana	MARE	Laticauda	LATI
Modenese	MDNS	Leccese	LECE
Modicana	MODC	Massese	MASS
Pezzata Rossa d’Oropa	PROR	Merinizzata	MERI
Piedmontese	PIDM	Pinzirita	PINZ
Pinzgau	PNZG	Sambucana	SAMB
Podolica	PODO	Sardinian Ancestral Black	SARB
Pontremolese	PONT	Sardinian White	SARW
Pustertaler	PUST	Sopravissana	SOPR
Reggiana	REGG	Valle del Belice	VALB
Rendena	REND		
Romagnola	ROMG		
Rossa siciliana	RSIC		
Sarda	SARD		
Sardo-Bruna	SBRU		
Sardo-Modicana	SMOD		
Varzese-ottonese	VOTT		

**Table 2:** Redundancy analysis from the model: climate = latitude + longitude + latitude × longitude. Analysis of variance (test of 999 permutations; d.f.: degrees of freedom).

Source	d.f.	Variance	F	Pr (>F)
Latitude	1	20.48	62.36	0.001
Longitude	1	0.23	0.71	0.473
Latitude:longitude	1	0.83	2.53	0.084
Residual	46	13.46		

**Table 3:** RDA of bovine genome along geography. Inertial partition.

Source	Inertia	% Inertia
Latitude	509.92	7.5
Longitude	255.51	3.7
Latitude:longitude	235.25	3.5
Total	6818.52	100

**Table 4:** RDA of ovine genome along geography. Partition of inertia.

Source	Inertia	% Inertia
Latitude	581.94	10.35
Longitude	453.56	8.07
Latitude:longitude	443.61	7.89
Total	5621.12	100

**Table 5:** Decomposition (percentage and *p*.values) of the inertia according to geography and climate (partialled out for geography).

	RDA Component	Cattle (Fst = 0.177)		Sheep (Fst = 0.144)	
		**% Inertia**	***p*** **-Value**	**%**	***p*** **-Value**
Geog (Lat*Long)	RDA1	7.5	0.03	11.5	< 0.01
	RDA2	4.6	0.66	8.1	0.02
	RDA3	2.6	0.97	6.7	0.14
	Total	14.7		26.3	
Clim|Geog	RDA1	6	0.09	7.5	0.18
	RDA2	4.1	0.56	4.8	0.74
	Total	10.1		12.3	

## Data Availability

Climatic data were downloaded from the climond.org database as described in Material and Methods. 50 k SNP genotype data for the cattle breeds can be found at the following link: https://osf.io/vh72y/?view_only=8f9b5fc86ffa4835adf4bb2df1543ab8, accessed on 13 May 2022. 50 k SNP genotype data for the sheep breeds may be available upon request to the corresponding author.

## Supplementary Materials

The following supporting information can be downloaded at: https://www.mdpi.com/article/10.3390/ani12172198/s1. Figure S1: climatic variable loadings for the first axis from PCA on climatic data; Figure S2: climatic variable loadings for the second axis from PCA on climatic data; Table S1: 35 climatic covariables (from Bio01 to Bio35) extracted from the Climond database (https://www.climond.org/, accessed on 13 May 2022) with resolution 30’: 11 variables on temperature, and 8 variables each for moisture, radiation, and precipitation.
