# Supplementary material for: How Geography and Climate Shaped the Genomic Diversity of Italian Local Cattle and Sheep Breeds
Source: Animals (Basel). 2022 Aug 26;12(17):2198. doi: 10.3390/ani12172198 (PMC9454691; doi:10.3390/ani12172198)
Supplement: Supplementary file 1 [file animals-12-02198-s001.zip › supplementary_table_S1.pdf]

Variable Number	Variable	abbrev.
Bio01	Annual mean temperature	temp_mean
Bio02	Mean diurnal temperature range (mean(period max-min))	temp_rangD
Bio03	Isothermality (Bio02 – Bio07)	temp_iso
Bio04	Temperature seasonality (C of V)	temp_season
Bio05	Max temperature of warmest week	temp_max0
Bio06	Min temperature of coldest week	min_temp0
Bio07	Temperature annual range (Bio05-Bio06)	temp_rangY
Bio08	Mean temperature of wettest quarter	temp_wet
Bio09	Mean temperature of driest quarter	temp_dry
Bio10	Mean temperature of warmest quarter	temp_warm
Bio11	Mean temperature of coldest quarter	temp_cold
Bio12	Annual precipitation (mm)	rain_mean
Bio13	Precipitation of wettest week (mm)	rain_max0
Bio14	Precipitation of driest week (mm)	rain_min0
Bio15	Precipitation seasonality (C of V)	rain_season
Bio16	Precipitation of wettest quarter (mm)	rain_wet
Bio17	Precipitation of driest quarter (mm)	rain_dry
Bio18	Precipitation of warmest quarter (mm)	rain_warm
Bio19	Precipitation of coldest quarter (mm)	rain_cold
Bio20	Annual mean radiation (W m-2)	rad_mean
Bio21	Highest weekly radiation (W m-2)	rad_max0
Bio22	Lowest weekly radiation (W m-2	rad_min0
Bio23	Radiation seasonality (C of V)	rad_season
Bio24	Radiation of wettest quarter (W m-2)	rad_wet
Bio25	Radiation of driest quarter (W m-2)	rad_dry
Bio26	Radiation of warmest quarter (W m-2)	rad_warm
Bio27	Radiation of coldest quarter (W m-2)	rad_cold
Bio28	Annual mean moisture index	moist_mean
Bio29	Highest weekly moisture index	moist_max
Bio30	Lowest weekly moisture index	moist_min

	Bio31	Moisture index seasonality (C of V)	moist_season
	Bio32	Mean moisture index of wettest quarter	moist_wet
	Bio33	Mean moisture index of driest quarter	moist_dry
	Bio34	Mean moisture index of warmest quarter	moist_warm
	Bio35	Mean moisture index of coldest quarter	moist_cold
