# Supplementary figures and images for: How Geography and Climate Shaped the Genomic Diversity of Italian Local Cattle and Sheep Breeds
Source: Animals (Basel). 2022 Aug 26;12(17):2198. doi: 10.3390/ani12172198 (PMC9454691; doi:10.3390/ani12172198)

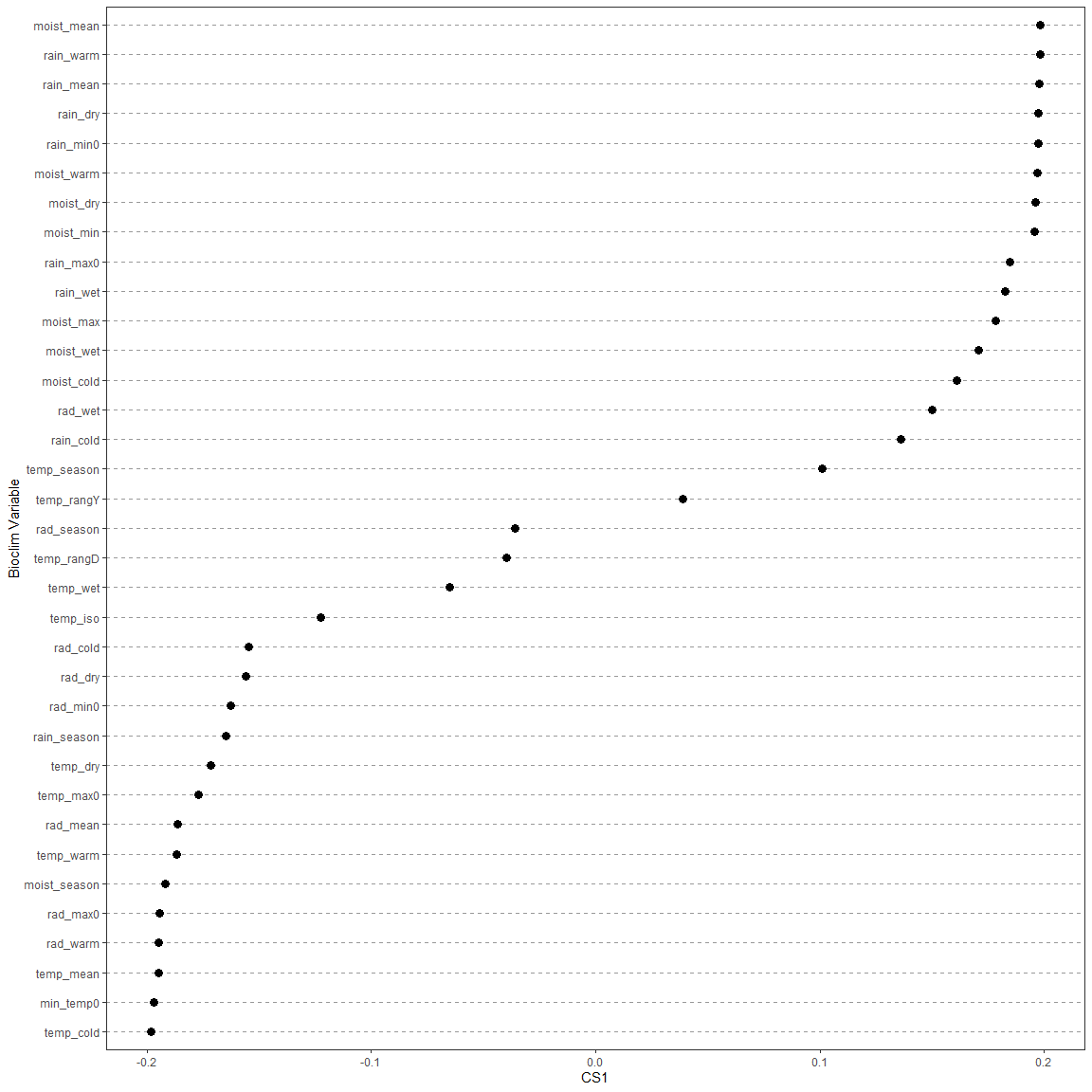

Supplement: Supplementary file 1 [file animals-12-02198-s001.zip › Supplementary_figure_S1.png]

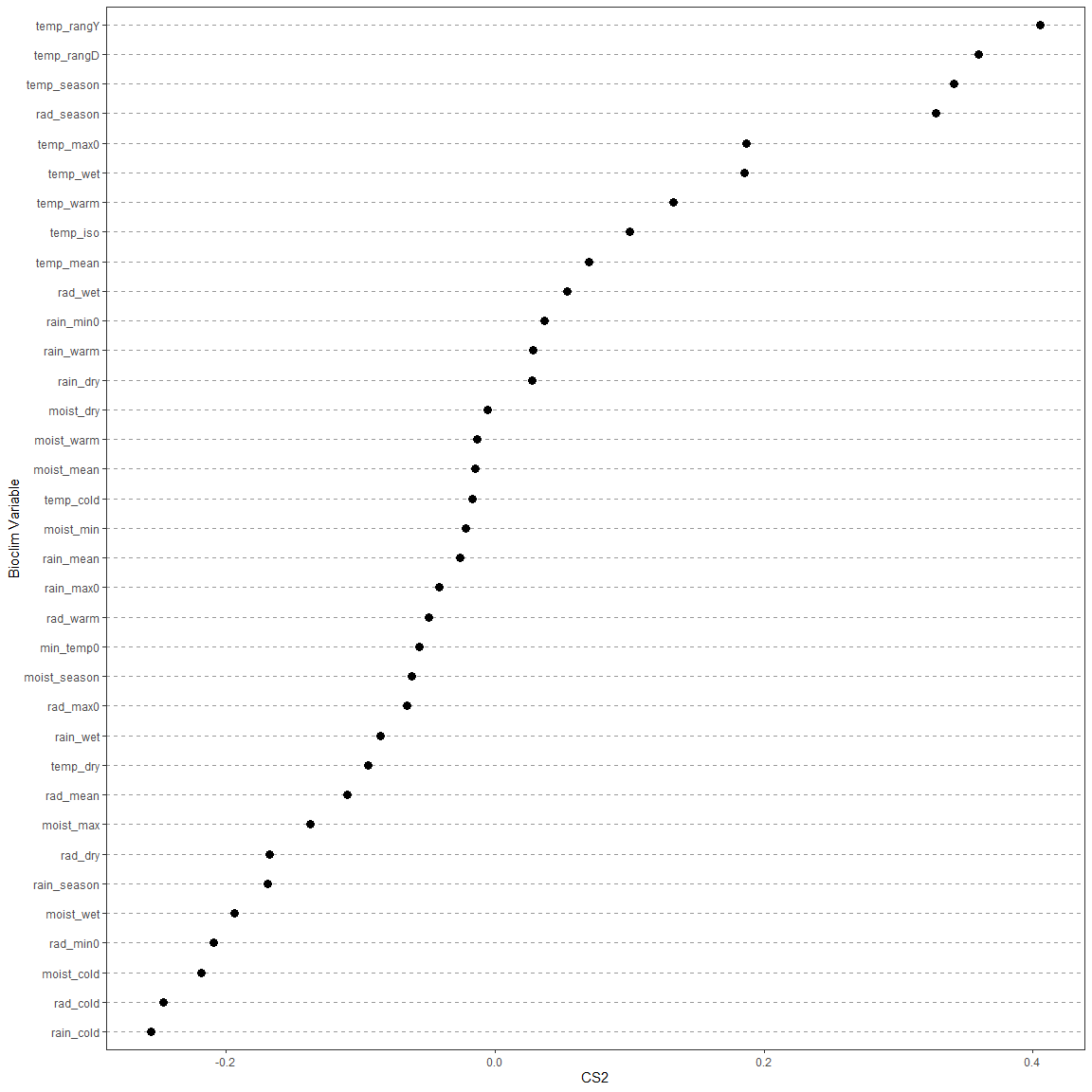

Supplement: Supplementary file 1 [file animals-12-02198-s001.zip › Supplementary_figure_S2.png]
